# Posterior vitreous detachment and macular microvasculature in the elderly

**DOI:** 10.1371/journal.pone.0231351

**Published:** 2020-04-08

**Authors:** Taku Toyama, Hisashi Kawai, Tomoyasu Shiraya, Fumiyuki Araki, Koichiro Sugimoto, Yohei Hashimoto, Satoshi Kato, Jiro Numaga, Yutaka Watanabe, Hirohiko Hirano, Yoshinori Fujiwara, Kazushige Ihara, Hunkyung Kim, Shuichi Obuchi, Takashi Ueta

**Affiliations:** 1 Department of Ophthalmology, Graduate School of Medicine and Faculty of Medicine, The University of Tokyo, Tokyo, Japan; 2 Tokyo Metropolitan Institute of Gerontology, Tokyo, Japan; 3 Department of Oral Health Science, Gerodontology, Faculty of Dental Medicine, Hokkaido University, Sapporo, Japan; 4 Department of Social Medicine, Hirosaki University School of Medicine, Hirosaki, Japan; 5 Department of Ophthalmology, National Center for Global Health and Medicine, Shinjuku City, Japan; Massachusetts Eye & Ear Infirmary, Harvard Medical School, UNITED STATES

## Abstract

**Purpose:**

To investigate the association between different stages of posterior vitreous detachment (PVD) and macular microvasculature in the elderly.

**Methods:**

Swept-source optical coherence tomography (OCT), OCT angiography, and color fundus images of 490 eyes without retinal pathologies of 322 participants aged ≥65 years were evaluated. PVD was classified using enhanced vitreous visualization mode as no apparent PVD (stage 0/1), vitreous adhesions at the fovea and optic disc (stage 2), adhesion at the optic disc (stage 3), or complete PVD (stage 4). Microvascular parameters, including foveal avascular zone (FAZ) and vessel density (VD), were analyzed for their associations with complete PVD. Additionally, the association between PVD and central retinal thickness (CRT) was also addressed.

**Results:**

Overall, 80, 31, 31, and 349 eyes were categorized into stages 0/1, 2, 3, and 4, respectively. Using multivariate mixed-effects model, the mean superficial FAZ area was smaller in stage 4 compared with stages 0–3 (0.29 vs. 0.32 mm^2^; *P* = 0.014), and the mean superficial VD was lower in stage 4 compared with stages 0–3 (34.96% vs. 35.24%; *P* = 0.0089). However, PVD was not significantly associated with deep macular microvascular parameters or CRT.

**Conclusions:**

Complete PVD was associated with smaller FAZ area and lower VD in superficial macular microvasculature, while it was not associated with central retinal thickness.

## Introduction

Posterior vitreous detachment (PVD) is one of the most common physiological phenomena experienced by the middle-aged and elderly populations. It manifests as vitreous gel liquefaction and weakened vitreoretinal adhesion[1]. It produces the symptoms of flashes and floaters in the visual field and plays a key role in the pathogenesis and prognosis of diverse vitreoretinal interface disorders, including macular holes[2], epiretinal membrane[3], vitreomacular traction syndrome[2], retinal detachment[4], diabetic retinopathy[5], and age-related macular degeneration (AMD)[6]. Therefore, concerning the pathology that occurs at the vitreoretinal interface, discussing PVD is integral. It can be observed via slit lamp biomicroscopy and indirect lenses in daily clinical practice. However, recent advances in optical coherence tomography (OCT), especially swept-source OCT (SS-OCT) with enhanced vitreous visualization mode, have enabled detailed and precise evaluation of the vitreoretinal interface[7].

Optical coherence tomography angiography (OCTA) is based on motion contrast imaging of high-resolution volumetric blood flow data. It allows for the generation of angiographic image slices of the retina as well as non-invasive three-dimensional vascular mapping of retinal microcirculation at different retinal depths. Recently, OCTA has been evaluated in clinical studies, proving particularly useful in those on retinal diseases[8]. Images taken by OCTA have been reported to be associated with the severity and clinical course of diabetic retinopathy (DR)[9], retinal vein occlusion (RVO)[10], retinopathy of prematurity[11], and AMD[12].

The purpose of the current study is to investigate whether or not there is an association between PVD evaluated with SS-OCT and macular microvascular parameters obtained via OCTA among the community-dwelling elderly population in a metropolitan area of Tokyo.

## Methods

### Participants

This cross-sectional study was conducted in 2018 as part of a prospective cohort study enrolling the community-dwelling elderly population in the Itabashi ward, a northwestern area of Tokyo. The cohort study, which had been conducted since 2011 and called “OTASSHA-KENSHIN” (meaning medical checkup for wellness of the elderly), was approved by the institutional review board and ethics committee of the Tokyo Metropolitan Institute of Gerontology[13,14], and conducted in adherence to Declaration of Helsinki.

In the OTASSHA-KENSHIN study, community-dwelling volunteers aged 65 years or older annually underwent comprehensive clinical examinations, including hematology tests, body composition measurements, cognitive function tests, and comorbidity and social background questionnaires. In 2018, in addition to these annual comprehensive examinations, the participants underwent fundus color photography, OCT, and OCTA with experienced optometrists who were not aware of the purpose of the present study.

For the current study, the exclusion criteria were as follows: 1) eyes with a history of retinal disease (i.e., DR, RVO, AMD, glaucoma, myopic maculopathy, and epiretinal membrane), as reported by the participants through a questionnaire or demonstrated by fundus photos, OCT, or OCTA obtained through the current study; 2) images with insufficient quality, as measured by a built-in software (i.e., an intelligence quotient (IQ) value of 40 or less)[9]; and 3) OCTA images with motion or shadow artifacts.

### OCT/OCTA image acquisition

The participants underwent OCT/OCTA using an SS-OCT (DRI OCT Triton; Topcon Inc., Tokyo, Japan) with a wavelength of 1,050 nm, an acquisition speed of 100,000 A-scans per second, and an axial and transversal resolution of 7 and 20 μm, respectively, in tissue. Two trained operators captured standard three-dimensional 4.5 mm × 4.5 mm macular cubes for each eye. Slabs of the superficial capillary plexus (SCP) and deep capillary plexus (DCP) were automated and segmented by the built-in software (IMAGEnet6, v1.23.15008, Basic License 10). The SCP was delineated from 2.6 μm below the internal limiting membrane to 15.6 μm below the junction between the inner plexiform and inner nuclear layers, and the DCP was delineated from 15.6 μm below the inner plexiform and the inner nuclear layers to 70.2 μm below them[15].

### PVD stage and central retinal thickness (CRT)

**Fig 1:**
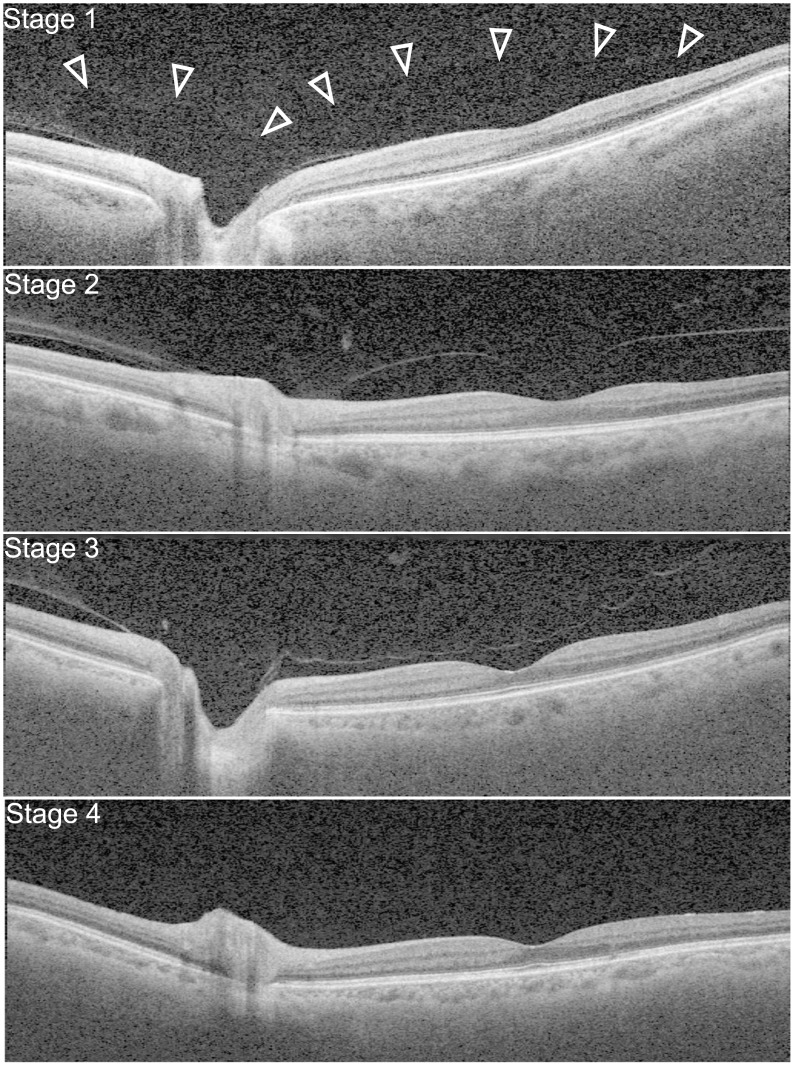
PVD stages observed in the elderly participants. In stage 1, adherent vitreous with premacular bursa and area of Martegiani (arrowhead) is observed. In stage 2, PVD occurs except persistent adhesion to the papilla and fovea. In stage 3, PVD occurs except persistent adhesion to the papilla. In stage 4 complete PVD was observed.

The SS-OCT images covered the posterior retina, including the macula and optic nerve disc (7 mm × 7 mm area centered on the fovea). The extent of PVD was graded based on a recently proposed system[7]: stage 0 = no PVD; stage 1 = PVD at mid-periphery and possible subtle PVD in the posterior retina; stage 2 = PVD, except for persistent adhesion to the papilla and fovea; stage 3 = PVD, except for persistent adhesion to the papilla; and stage 4 = complete PVD. The staging was based on a recent study that montaged SS-OCT images covering the midperipheral fundus[7]. The same study also showed that in most elderly people over 60 years old, PVD is at stage 1 or higher[7]. In the current study, SS-OCT images of the posterior retina were obtained, and we did not differentiate between PVD stages 0 and 1; hence, eyes were categorized into PVD stages 0/1, 2, 3, and 4 (Fig 1).

CRT has been known to be associated with FAZ area[16]. We addressed the association between PVD and CRT that was defined as the mean retinal thickness within 1mm ETDRS circle on macular thickness map.

### OCTA image analysis

Using the ImageJ software (National Institute of Health, Bethesda, Maryland, U.S.A.), the grayscale OCTA images were standardized and cropped (3 mm × 3 mm), with the fovea at the center.

**Fig 2:**
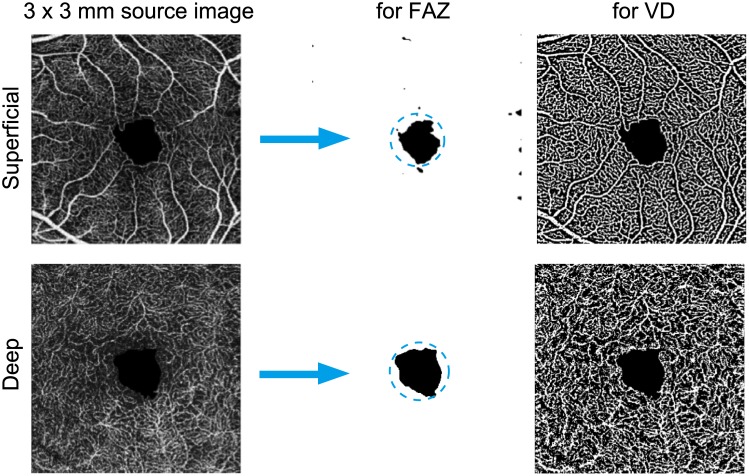
OCTA Image processing for FAZ and VD analyses. FAZ is the black area indicated inside the dotted circle. For VD calculation, FAZ area was subtracted from the total area, then white/black area was calculated.

The ImageJ macro for the FAZ measurement was as follows: Each image was edited in the order of smooth Gaussian Blur 3D (X sigma = 2.0, Y sigma = 2.0, and Z sigma = 2.0) and Auto Local Threshold minimum (Fig 2). Then, using ImageJ, the FAZ was binarized and extracted, and the area, perimeter, and circularity index of the FAZ were measured automatically. The FAZ perimeter refers to the circumference of the FAZ. The FAZ circularity index shows how close the shape of the FAZ is to a perfect circle; the closer the value is to 1.0, the higher the circularity is.

For the VD measurement, using ImageJ, each image was binarized via the Niblack method (Fig 2), and the VD was calculated as follows: vessel area/(3 mm × 3 mm − FAZ area)[16].

### Statistics

A statistical analysis was performed using the JMP Pro 14 software (SAS). For the univariate analysis, the ANOVA and Fisher's exact test were used to compare continuous and categorical data, respectively, among the different PVD stages. To include both eyes of the same participant in the analysis, a mixed model was applied for the multivariate analysis. The effects of age, sex, diabetes mellitus (DM), hypertension, and smoking status (i.e., current, past, or never) were adjusted to explore the possible effect of PVD on retinal microvascular parameters. The summarized continuous data were presented as mean ± standard deviation (SD). A *P*-value of <0.05 was regarded as statistically significant.

## Results

Images of 490 eyes (322 participants) were judged eligible for analyses in the present study. Among the 490 eyes included, 80 (16.3%), 30 (6.1%), 31 (6.3%), and 349 (70.9%) were categorized into PVD stages 0/1, 2, 3, and 4, respectively. Table 1 summarizes the characteristics stratified by PVD stage. The univariate analysis did not detect a significant difference among the distributions of sex, DM, and dyslipidemia across the different PVD stages. Significant differences were detected in age and hypertension through the ANOVA and Fisher’s exact test, respectively; however, pairwise comparisons using the Bonferroni correction did not reveal a significant difference between any specific pairs. The OCT image quality, represented as IQ value, was equivalent across the different PVD stages.

**Table 1:** Characteristics of participants stratified by PVD stage.

	Stage 0/1	Stage 2	Stage 3	Stage 4	*P* value
Eyes, n	80	30	31	349	
Age, mean±SD	69.5±4.97	68.0±2.54	70.1±6.11	70.73±5.83	0.033
Sex,n(%)					
male	44(55.0%)	10(33.3%)	10(32.3%)	148(42.4%)	0.67
female	36(45.0%)	20(67.7%)	21(67.7%)	201(57.6%)	
DM, n(%)	8(10.0%)	5(16.7%)	4(12.9%)	38(10.9%)	0.69
Hypertension, n(%)	37(46.3%)	6(20.0%)	15(48.4%)	118(33.8%)	0.021
Dyslipidemia, n(%)	36(45.0%)	6(46.7%)	15(29.0%)	122(35.0%)	0.19
IQ, mean±SD	65.4±0.73	65.8±1.2	65.9±1.2	65.5±0.35	0.98

DM; diabetes mellitus. IQ; OCTA image quality metrics. SD; standard deviation.

Next, we investigated the effect PVD on macular microvascular parameters. As the number of eyes with PVD stage 2 or 3 was relatively small, we compared the effects of PVD stages 0–3 (i.e., no or incomplete PVD) with those of stage 4 (i.e., complete PVD). After adjusting for the effects of age, sex, and history of DM, hypertension, and smoking status, stage-4 PVD was significantly associated with a smaller superficial FAZ area compared with stages 0–3 (0.32 vs. 0.29 mm^2^, *P* = 0.014; Table 2). In addition, stage-4 PVD was significantly associated with a lower superficial VD compared with stages 0–3 (35.24% vs. 34.96%, *P* = 0.0089; Table 2). There was no significant effect of PVD on the superficial FAZ perimeter, superficial FAZ circularity, or any of the parameters related to deep macular microvasculature (Table 2).

**Table 2:** Effects of complete PVD (stage 4) on macular microvascular parameters compared to earlier PVD stages (stage 0–3).

	Stage 0–3 (n = 141)	Stage 4 (n = 349)	Estimate (95%CI)	*P* value
Superficial				
FAZ area, mm^2^	0.32±0.12	0.29±0.11	0.0139 (0.00283 to 0.0249)	0.014*
FAZ perimeter, mm	2.36±0.51	2.27±0.50	0.0472 (-0.0115 to 0.106)	0.11
FAZ circularity index	0.71±0.10	0.70±0.10	0.00326 (-0.00887 to 0.0154)	0.60
Vessel density, %	35.24±0.81	34.96±0.75	0.126056 (0.0319 to 0.220)	0.0089*
Deep				
FAZ area, mm^2^	0.47±0.26	0.42±0.24	0.0167 (-0.00993 to 0.0433)	0.22
FAZ perimeter, mm	3.16±1.33	2.99±1.32	0.0672 (-0.0798 to 0.214)	0.37
FAZ circularity index	0.61±0.15	0.61±0.16	-0.00148 (-0.0956 to 0.0166)	0.87
Vessel density, %	35.10±1.17	34.87±1.20	0.0924 (-0.0348 to 0.220)	0.15

FAZ; foveal avascular zone. CI; confidence interval.

*P indicates statistical significance (< 0.05).

Adjusted for age, sex, history of DM and hypertension, and smoking status.

To confirm the robustness of the conclusion, we compared the effects of PVD stage 0/1 (i.e., no or subtle PVD) with those of stage 4 (i.e., complete PVD) on macular microvascular parameters. Again, compared with stage 0/1, stage-4 PVD was associated with a smaller superficial FAZ area (0.31 vs. 0.29 mm^2^, *P* = 0.048; Table 3) and lower superficial VD (35.31% vs. 34.96%, *P* = 0.0038; Table 3) but not associated with superficial FAZ perimeter/circularity or deep retinal microvascular parameters (Table 3).

**Table 3:** Effects of complete PVD (stage 4) on macular microvascular parameters compared to no apparent PVD (stage 0/1).

	Stage 0/1 (n = 80)	Stage 4 (n = 349)	Estimate (95%CI)	*P* value
Superficial				
FAZ area (mm^2^)	0.31±0.11	0.29±0.11	0.0154 (0.000152 to 0.0306)	0.048*
FAZ perimeter(mm)	2.27±0.46	2.27±0.50	0.0266 (-0.0492 to 0.102)	0.49
FAZ circularity index	0.72±0.10	0.70±0.10	0.00719 (-0.00851 to 0.0229)	0.37
Vessel density(%)	35.31±0.72	34.96±0.75	0.178 (0.0581 to 0.298)	0.0038*
Deep				
FAZ area(mm^2^)	0.45±0.27	0.42±0.24	0.0122 (-0.0220 to 0.0464)	0.48
FAZ perimeter(mm)	3.10±1.41	2.99±1.32	0.0603 (-0.131 to 0.252)	0.54
FAZ circularity index	0.61±0.16	0.61±0.16	-0.00185 (-0.0255 to 0.0218)	0.88
Vessel density(%)	35.12±1.15	34.87±1.20	0.122 (-0.0398 to 0.283)	0.14

FAZ; foveal avascular zone. CI; confidence interval.

*P indicates statistical significance (< 0.05).

Adjusted for age, sex, history of DM and hypertension, and smoking status.

From the mechanistic viewpoint, we speculated that PVD could influence the macular microvascular parameters through its effect on central retinal thickness (CRT), because CRT is associated with FAZ area in healthy eyes[16]. Therefore, we addressed whether PVD could influence CRT. The mean±SD CRT of eyes with PVD stage 0–3, stage 0/1, and stage 4 were 231.9±24.1, 234.5±25.3, and 233.5±21.5 μm, respectively. After adjusting for the effects of age and sex, there was no significant difference in CRT between PVD stage 0–3 vs. stage 4 (estimate [95% CI] = 0.25[-1.40–1.89], *P* = 0.77) or between PVD stage 0 vs. stage 4 (estimate [95% CI] = -0.24[-4.67–4.18], *P* = 0.91). The results did not support the hypothesis that CRT is involved in the effect of PVD on macular microvascular parameters.

## Discussion

The present study investigated the association between PVD and retinal microvasculature via SS-OCT and SS-OCTA among the community-dwelling elderly in Tokyo. Our results suggest that PVD has effects on superficial retinal microvasculature in the elderly, as demonstrated by a smaller FAZ area and lower macular VD. However, PVD does not appear to have a significant effect on deep macular microvasculature or CRT.

In previous studies, correlations of retinal microvasculature to demographic, systemic, and retinal diseases have been extensively studied using OCTA. For example, females have a larger FAZ area in normal eyes[17]. In addition, DM, even without DR, can affect macular microvascular parameters, e.g., an enlarged FAZ area[18] and decreased VD[19]. Hypertension has also been suggested to be associated with a larger FAZ area and lower VD[20]. Moreover, in retinal vascular diseases, including DR, retinal vein occlusion, and retinopathy of prematurity, the disease severity and clinical prognosis correlate with changes in the microvascular parameters on OCTA[9–11]. In contrast, the effect of age on retinal microvasculature remains controversial. Coscas et al. found that the superficial FAZ size as well as both the superficial and deep VD is significantly decreased in older subjects[21]. In contrast, Iafe et al[22]. and Yu et al[23]. reported a significantly larger FAZ and lower VD in older subjects. However, other studies have not observed a significant effect of age on retinal microvasculature on OCTA[17,24,25]. Since age is the factor most closely associated with PVD development[7] and our data suggest that PVD influences the superficial FAZ and VD, the ongoing discussion about the effects of age on FAZ and VD may need to be reevaluated with the inclusion of PVD status as a confounding factor.

Our conclusion that PVD is associated with a smaller FAZ area and lower VD was consistent across two comparisons, one comparing PVD stages 0–3 with stage 4 and the other comparing stage 0/1 with stage 4. We speculated how PVD could confer such effects on the superficial macular microvasculature. According to a recent report in which PVD development was investigated on montaged SS-OCT images, a subtle separation of the vitreous cortex from some parts of the retina has started (called stage Ib) in most of the elderly[7], and PVD stages 0 or 1a are considered relatively rare in the population aged 65 years old or older[7]. Thus, we speculate that the vitreous adhesion may produce centrifugal tangential force in the posterior retina, leading to a larger FAZ size in eyes without PVD compared with those with PVD. When PVD is complete, the posterior retina will be free of the tangential force of the vitreous, resulting in a smaller FAZ area.

In addition to physical changes in the tangential force on the retina, PVD is accompanied by autoregulatory physiological changes. Previous reports have revealed that the partial pressure of oxygen in the vitreous increases in the presence of PVD[26], by which the retinal oxygenation is also expected to be higher[27]. In general, tissue oxygenation is tightly regulated by vasodilation/vasoconstriction that is mediated by nitric oxide (NO)[28], an important mediator in response to lower oxygenation that dilates vessels to restore tissue oxygenation. Therefore, in elderly eyes without PVD, there could be lower retinal oxygenation, which could lead to relative vasodilation, represented as a higher VD value. In contrast, in eyes with PVD, there could be higher retinal oxygenation, which could prevent NO from enhancing vasodilation and lead to a lower VD in eyes with PVD, as observed in the current study. Autoregulatory response of the retinal capillaries has also been suggested in diabetic eyes without DR[29].

Furthermore, the change in tangential force and retinal oxygenation induced by PVD is considered to affect the inner retina more than the outer retina, which is consistent with our results that only superficial macular microvascular parameters are influenced by PVD.

Several limitations exist in this study. To start, the enrolled participants were community-dwelling elderly volunteers whose medical and social backgrounds were not uniform. We performed multivariate analyses adjusted for age, sex, history of DM and HT, and smoking status to carefully address the possible effects of PVD on retinal microvasculature evaluated via SS-OCT. However, current understanding about the factors affecting OCTA-imaged retinal microvasculature is imperfect, and we could not adjust for unexpected confounding factors. In addition, the present study was not performed based on detailed ophthalmic examinations; medical history was collected through questionnaires, and fundus color, OCT, and OCTA images were carefully examined to detect pre-existing retinal pathologies. We could not collect data on refractive error or axial length, which might have caused some errors in the FAZ area estimates[24]. However, a recent study argued that axial length does not have a significant effect on the FAZ[30]. As a strength of the current study, 490 eyes, a substantially larger number of eyes compared with those in previous studies, were included for OCTA image analysis, which should have improved the accuracy of our conclusion. Ocular media opacity in the elderly population might have affected the OCTA image quality. However, we carefully excluded low-quality images using the IQ threshold of 40, and equivalent image quality was confirmed across the different PVD stages.

In summary, the association between PVD and macular microvasculature imaged via SS-OCT and SS-OCTA was investigated in the community-dwelling elderly in Tokyo. We observed a consistent association between complete PVD with a smaller FAZ area and lower VD in the superficial macula but not in the deep macular microvasculature. Our data, strengthened by the inclusion of 490 eyes, suggest an impact of PVD on macular microvasculature in the elderly.
